# Collateral damage: Fear from SARS-CoV2-infection causing Takotsubo cardiomyopathy

**DOI:** 10.1007/s00392-020-01706-w

**Published:** 2020-07-13

**Authors:** Tobias Uhe, Andreas Hagendorff, Rolf Wachter, Ulrich Laufs

**Affiliations:** Klinik und Poliklinik für Kardiologie, Universitätsklinikum Leipzig, Leipzig, Germany

Sirs:

An 84-year-old male patient with known ischemic cardiomyopathy was admitted to the emergency department of Leipzig University Hospital with typical signs and symptoms of an acute coronary syndrome in the midst of the SARS-Cov2 pandemic on April 22, 2020. His angina rapidly resolved after angioplasty of a sub-total proximal occlusion of his right coronary artery. However, 12 h later his 81-year-old wife was brought to a secondary care hospital by the emergency doctor because of acute and severe shortness of breath and typical angina. Elevated Troponin, ECG- and echocardiographic abnormalities led to her urgent transfer to our hospital with the suspected diagnosis of acute myocardial infarction .

**Table 1:** Laboratory results on admission

		Reference range
CK	1.79 µkat/l	(0.43–2.34 µkat/l)
CK-MB	0.47 µkat/l	(< 0.42 µkat/l)
hs-Troponin	482.0 ng/l	(< 14.0 ng/l)
Myoglobin	69.8 µg/l	(25–58 µg/l)
Creatinine	140 µmol/l	(45–84 µmol/l)
eGFR	30 ml/min/1.73 m^2^	(> 90 ml/min/1.73m^2^)
Urea	12.6 mmol/l	(< 11.9 mmol/l)
LDH	4.82 µkat/l	(2.25–3.55 µkat/l)

Her medical history included chronic renal insufficiency and hypertension. Otherwise, the patient was previously well, mobile and in very good general condition. Clinical examination showed an obese woman (163 cm, 97 kg, blood pressure 130/73 mmHg, heart rate 78/min, and body temperature 37.2 °C). The laboratory findings are presented in Table 1.

**Fig. 1:**
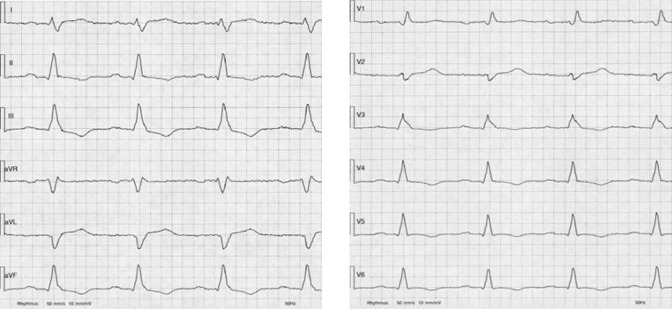
Electrocardiogram at admission

The 12-lead-ECG at admission displayed a sinus rhythm with a previously known right bundle branch block and new negative T-waves in II, III, aVF and V3-6 (Fig. 1).

**Fig. 2:**
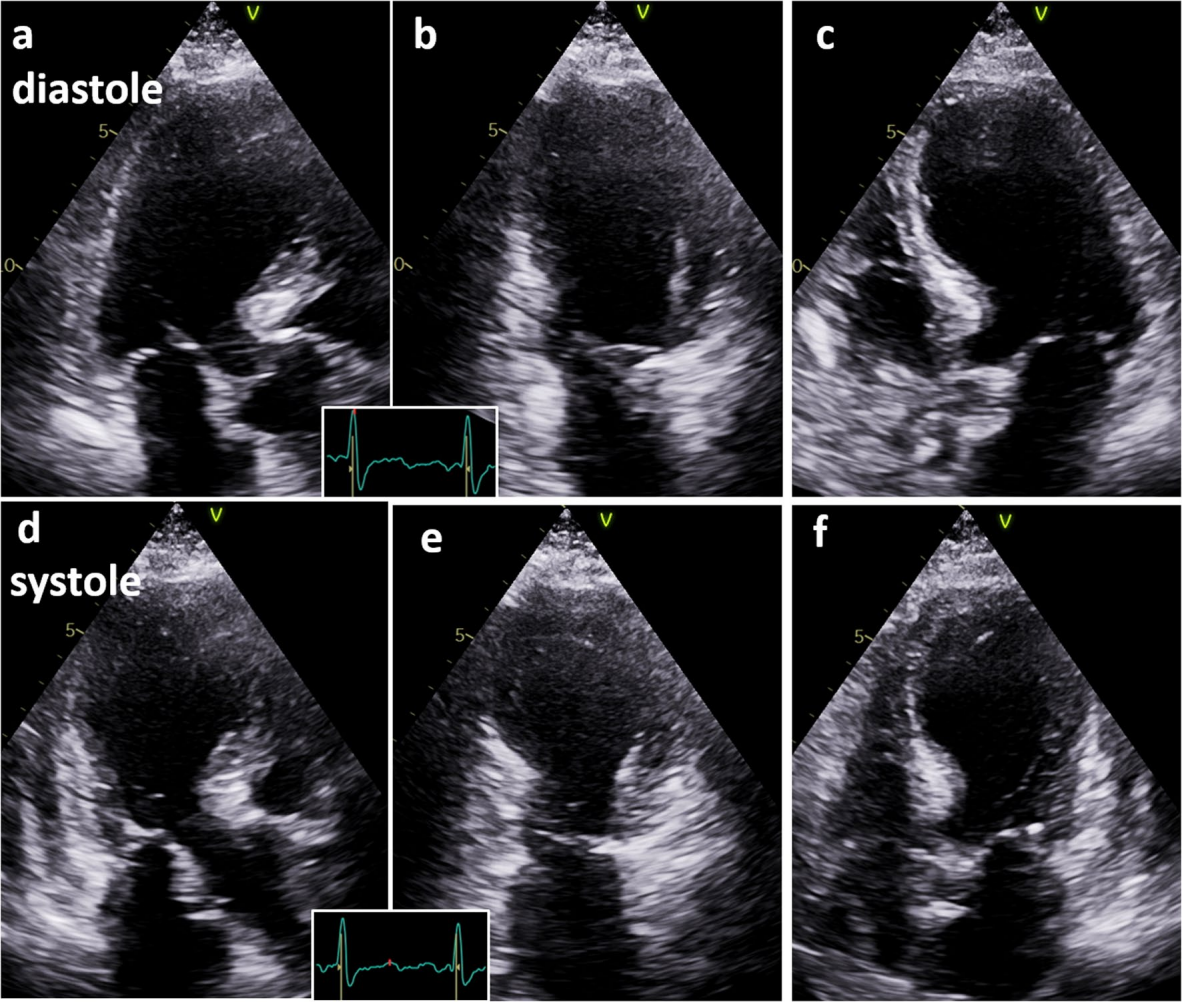
Echocardiography showed wall motion abnormalities with typical apical ballooning documented by apical long axis view, 2- and 4- chamber view during diastole (**a**-**c**) and systole (**d**-**f**)

**Fig. 3:**
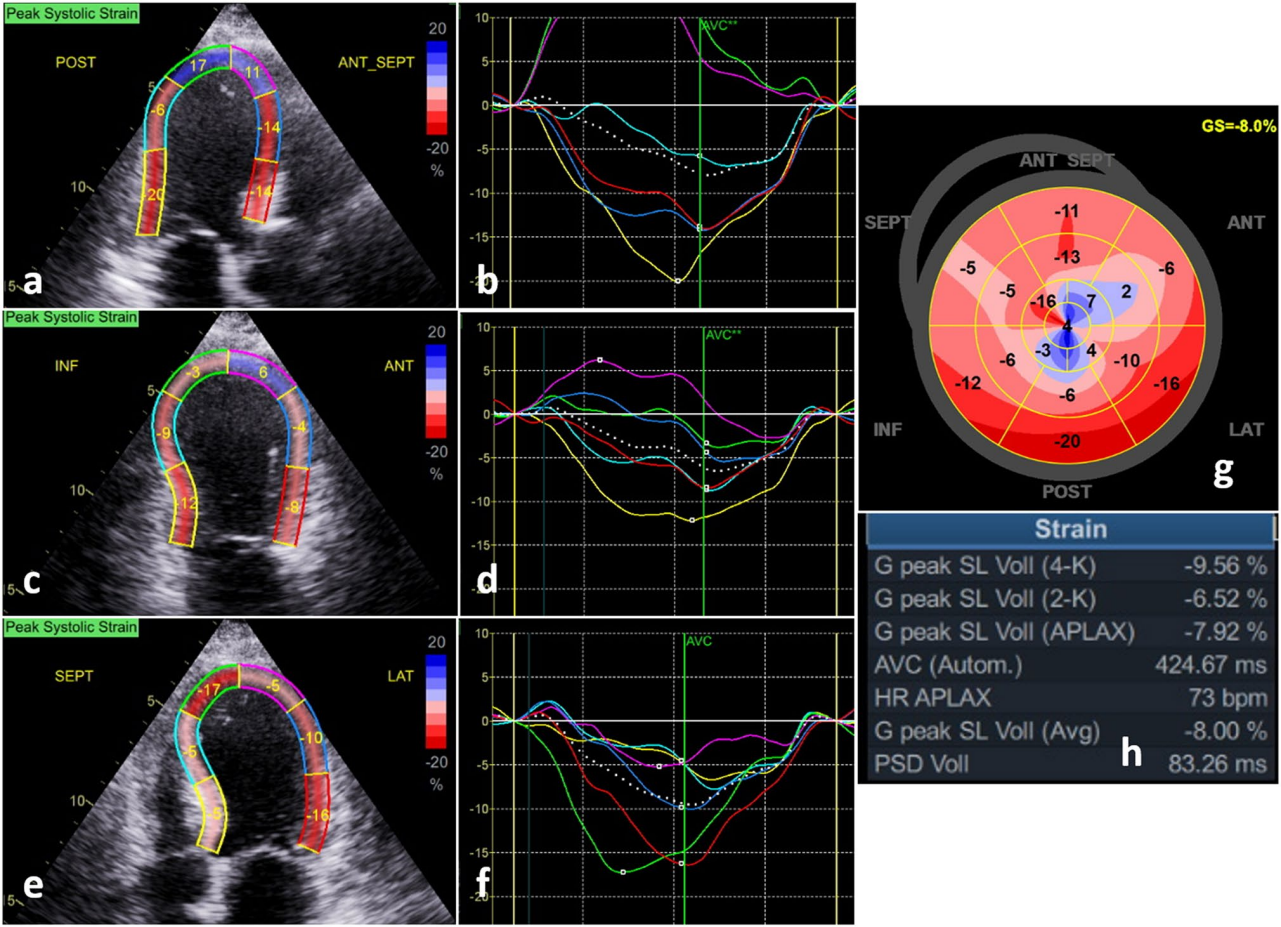
Echocardiographic deformation imaging at acute stage. Peak systolic strain and corresponding regional longitudinal strain curves tracked in the apical long axis view (**a**, **b**), the 2-chamber view (**c**, **d**) and the 4-chamber view (**e**, **f**). The bull’s eye plot of the typical pattern of regional longitudinal strain of the left ventricle is presented in g, the respective strain data are shown in h

Echocardiography (Fig. 2) showed wall motion abnormalities with apical septal dyskinesis, mid ubiquitous akinesia and basal septal and anterior hypokinesis. Left ventricular ejection fraction was reduced to 45%. Global longitudinal strain with a typical strain pattern of apical balloning was −8% (Fig. 3).

**Fig. 4:**
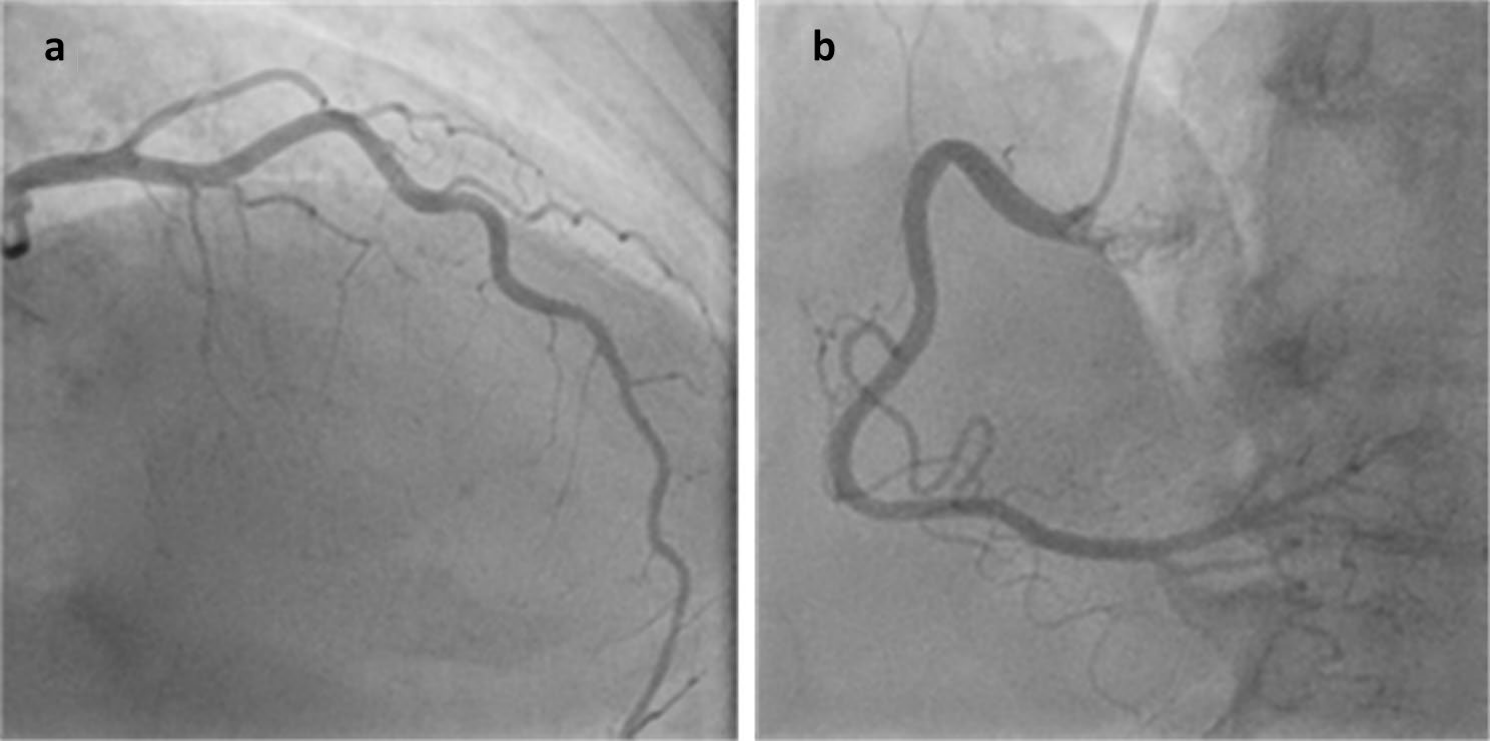
Coronary angiogram without signs of CAD in the left (**a**) and the right (**b**) coronary artery

Coronary angiogram revealed no signs of artery disease (CAD), as shown in Fig. 4.

**Fig. 5:**
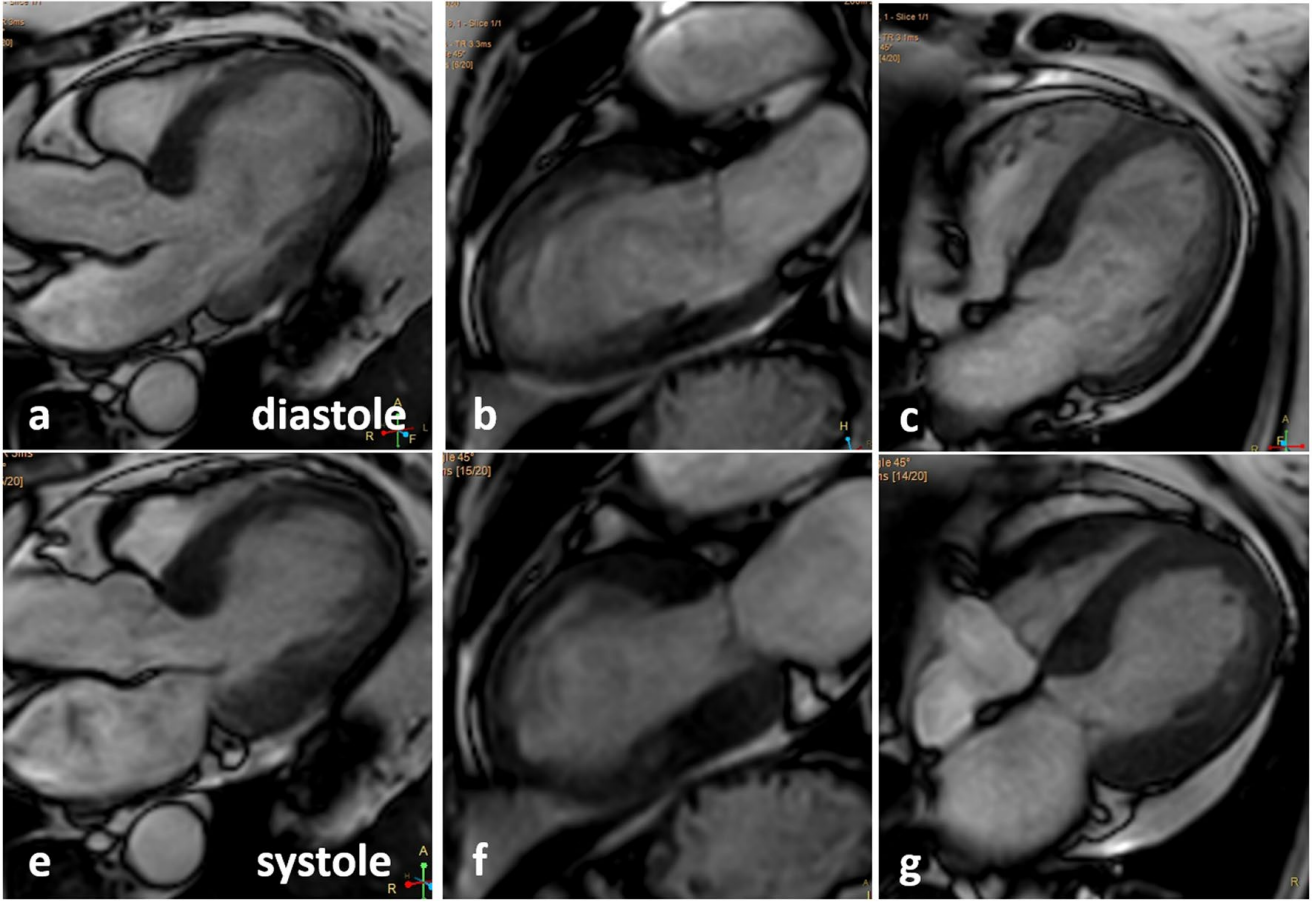
Cine B-TFE sequences of cardiac MR illustrate the wall motion abnormalities with typical apical ballooning documented by apical long axis view, 2- and 4- chamber view during diastole (**a**-**c**) and systole (**d**-**f**)

**Fig. 6:**
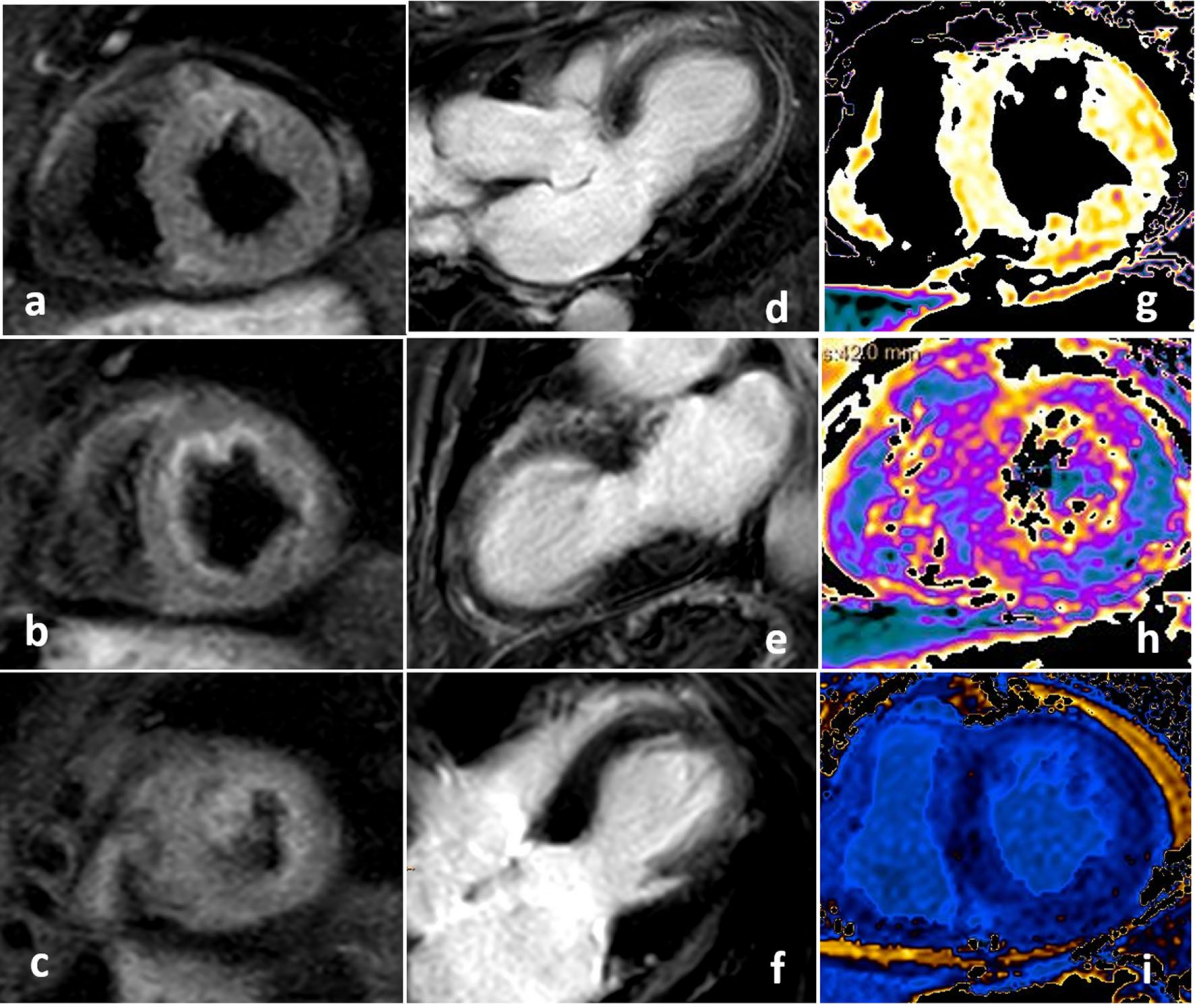
MR documentation of the ubiquitous edema by T2STIR sequences in basal, mid and apical short axis views (**a**-**c**); documentation of diffuse late enhancement in T1TFE sequences in the long axis view, the 2- and 4-chamber view (**d**-**f**). Representative colored illustration of T1-mapping (**g**), T2-mapping (**h**) and T1-mapping after contrast (**i**) for assessment of edema and extracellular volume

Cardiac MR (Fig. 5) showed wall motion abnormalities corresponding to the echocardiography. The tissue characterization by T2STIR sequences documented severe edema (Fig. 6). T1TFE sequences showed diffuse late enhancement. T1- and T2-mapping showed severely increased T1 and T2 values (1660 ms and 68 ms), respectively. Extracellular volume was ~ 37%.

In summary, the findings fulfill the criteria of a Takotsubo (stress) cardiomyopathy [1].

Focused evaluation of potential emotional or physical triggers revealed that the patient suffered from psychologic stress during the last few weeks. Due to media reports, she was highly afraid of SARS-CoV2 infections for her husband and herself, because age and hypertension were communicated as high risk for a severe course of COVID-19. Furthermore, she felt severely affected by the lockdown measures. Her condition acutely and severely exacerbated when her husband was admitted to the hospital, which she felt would place him at a high risk of death, and because she was not allowed to visit him due to the SARS-CoV2-specific regulations.

The patient was provided with psychological support and the SARS-CoV2-negative couple was allowed to meet. Heart failure therapy including an angiotensin receptor blocker, beta blocker, mineraloreceptor antagonist and a loop diuretic was initiated and the symptoms of the patient improved. She was discharged home in stabilized condition.

**Fig. 7:**
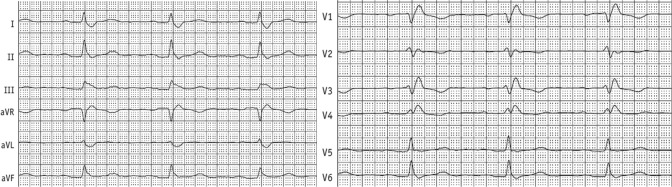
Electrocardiogram at follow-up

**Fig. 8:**
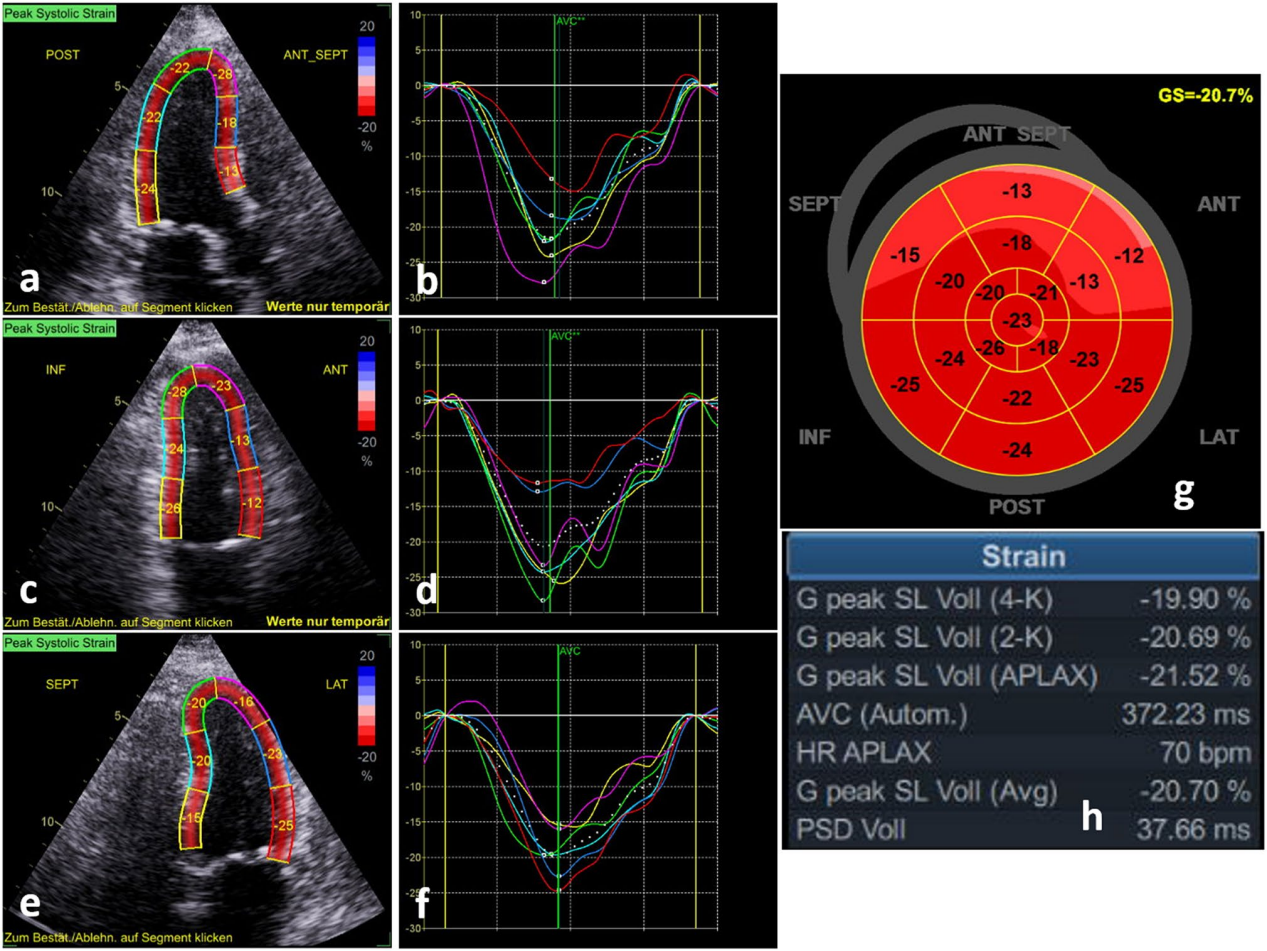
Follow-up echocardiographic deformation imaging after 2 months. Peak systolic strain and corresponding regional longitudinal strain curves tracked in the apical long axis view (**a**, **b**), the 2-chamber view (**c**, **d**) and the 4-chamber view (**e**, **f**). The bull’s eye plot of the typical pattern of regional longitudinal strain of the left ventricle is presented in g, the respective strain data are shown in (**h**)

Two months later, the patient presented to the outpatient clinic. She was asymptomatic and the negative T-waves in II, III and aVF had disappeared (Fig. 7). Echocardiography (Fig. 8) and cardiac MRI showed full recovery of left ventricular function.

Takotsubo (stress) cardiomyopathy (TTS) predominantly affects women. Approximately one-third of the cases are caused by emotional triggers and one-third by physical stress. Typical symptoms are angina pectoris or dyspnea; therefore, myocardial infarction is the most relevant differential diagnosis. Hypo- or akinesis of the apical and mid left-ventricular segments is characteristic. Additional common findings include transient ST-elevations or negative T-waves in ECG and increased Troponin. Increased catecholamine concentrations likely contribute to the pathophysiology that is still incompletely understood [1, 2]. Patients with TTS caused by emotional stress show better long-term outcomes compared with ACS patients or TTS with physical triggers [3].

This case is informative with regard to patient’s perceptions and an example for a collateral medical damage during the SARS-CoV2-pandemic. Collateral damages in this context range from patients avoiding contact to medical professionals despite symptoms, delayed or false diagnoses (because of changes in previously standardized processes) to indirect effects causing psychological and physical diseases [4–6]. The fear of infection but also misinformation can cause worsening of preexisting psychological disorders and potentially life-threatening conditions [7–10]. Careful education of patients at risk and information of the general population appears crucial to prevent these events.
